# Identification of New Regulators of the Oocyte-to-Embryo Transition in *Drosophila*

**DOI:** 10.1534/g3.120.401415

**Published:** 2020-07-20

**Authors:** Emir E. Avilés-Pagán, Albert S. W. Kang, Terry L. Orr-Weaver

**Affiliations:** * Department of Biology, MIT, 77 Mass Avenue, Cambridge MA 02138; † Whitehead Institute for Biomedical Research, 455 Main Street, Cambridge MA 02142

**Keywords:** mitosis, embryogenesis, oogenesis, F-box protein, glucose transporter

## Abstract

At the oocyte-to-embryo transition the highly differentiated oocyte arrested in meiosis becomes a totipotent embryo capable of embryogenesis. Oocyte maturation (release of the prophase I primary arrest) and egg activation (release from the secondary meiotic arrest and the trigger for the oocyte-to-embryo transition) serve as prerequisites for this transition, both events being controlled posttranscriptionally. Recently, we obtained a comprehensive list of proteins whose levels are developmentally regulated during these events via a high-throughput quantitative proteomic analysis of *Drosophila melanogaster* oocyte maturation and egg activation. We conducted a targeted screen for potential novel regulators of the oocyte-to-embryo transition, selecting 53 candidates from these proteins. We reduced the function of each candidate gene using transposable element insertion alleles and RNAi, and screened for defects in oocyte maturation or early embryogenesis. Deletion of the aquaporin gene *CG7777* did not affect female fertility. However, we identified *CG5003* and *nebu* (*CG10960*) as new regulators of the transition from oocyte to embryo. Mutations in *CG5003*, which encodes an F-box protein associated with SCF-proteasome degradation function, cause a decrease in female fertility and early embryonic arrest. Mutations in *nebu*, encoding a putative glucose transporter, result in defects during the early embryonic divisions, as well as a developmental delay and arrest. *nebu* mutants also exhibit a defect in glycogen accumulation during late oogenesis. Our findings highlight potential previously unknown roles for the ubiquitin protein degradation pathway and sugar transport across membranes during this time, and paint a broader picture of the underlying requirements of the oocyte-to-embryo transition.

The transition from oocyte to embryo marks the onset of development in most metazoans. In preparation for this transition, the developing oocyte undergoes a prolonged arrest in meiosis I that permits it to be endowed with maternally provided mRNAs, proteins and nutrients. Once this is accomplished, the oocyte resumes meiosis during maturation, then arrests at a second point in meiosis awaiting fertilization. Egg activation is the trigger for the oocyte-to-embryo transition, and it comprises a series of events that result in the completion of meiosis and the restoration of potency. Changes occurring during egg activation at the cellular level are accompanied by changes at the molecular level, namely changes in mRNA translation, protein levels, and redox (Avilés-Pagán and Orr-Weaver 2018; Schultz *et al.* 2018; Huelgas-Morales and Greenstein 2018). Egg activation does not require fertilization in Drosophila, instead relying on mechanical cues and calcium signaling set off by ovulation (Horner and Wolfner 2008b; Sartain and Wolfner 2013; Avilés-Pagán and Orr-Weaver 2018). The uncoupling of egg activation and fertilization in Drosophila, and its genetic tractability, allow for dissection of the regulatory mechanisms that underlie egg activation without confounding effects from expression of the zygotic genome and subsequent steps in embryonic development.

As the events of the oocyte-to-embryo transition occur in the absence of transcriptional activity in the oocyte (Kronja *et al.* 2014b), gene regulation is exclusively by posttranscriptional mechanisms and dependent on maternal stores of mRNAs and proteins. Regulation of the proteome through controlled protein synthesis, protein degradation and changes in post-translational modifications such as phosphorylation is crucial for proper progression through oocyte maturation and egg activation (Krauchunas *et al.* 2013; Kronja *et al.* 2014a; Guo *et al.* 2015; Zhang *et al.* 2018). Recent work characterized the proteome dynamics of oocyte maturation and egg activation in *Drosophila melanogaster*, uncovering different patterns of protein level changes and finding a contribution for both translation and proteolysis in shaping of the proteome during this time (Kronja *et al.* 2014a). In some instances, the pattern observed for a specific protein was predictive of its role in these developmental stages. Thus, these data provide a powerful resource for identifying potential new key regulators and relevant biological processes during oocyte maturation, egg activation and early embryogenesis.

We used the data on proteome dynamics during the oocyte-to-embryo transition as a basis to identify potential new regulators of this transition in Drosophila. Of particular interest were proteins whose changes in levels throughout this process suggested possible developmental regulation. We performed a targeted screen using a combination of germline-driven RNAi and available transposable element insertion alleles, and we identified several candidate genes whose disruption resulted in defects in late oogenesis or early embryogenesis. We further characterized *CG5003* and *nebu* (*CG10960*), two candidates of interest whose reduced function results in an embryonic mitotic phenotype and embryonic lethality. Moreover, we find a potential role for *nebu* in regulating glycogen metabolism at the oocyte-to-embryo transition. We undertook a parallel approach of knocking out a gene encoding a protein whose function suggested it should be required for egg activation, an aquaporin. This type of “biased” reverse genetics, selecting potential regulators based on known function, has limitations. We found there is no unique requirement for the oocyte-expressed aquaporin in egg activation.

## Materials and Methods

### Fly stocks

All flies were fed a cornmeal and molasses diet and kept at 18, 22 or 25°. *Oregon R* flies were used as a wild-type control for crosses involving transposable element insertions. The transposable element insertions were generated as part of Drosophila Gene Disruption Project. The RNAi lines from the Transgenic RNAi project (TRiP) were obtained from the Bloomington Drosophila Stock Center (BDSC); all other RNAi lines were obtained from the GD collection at the Vienna Drosophila Resource Center. The *matα4-GAL*, *UAS-Dicer2* chromosome was generated for this study by recombination of transgenes in the *maternal-tubulin*-Gal4 P{*matα4*-GAL-VP16}V37 driver stock and the *UAS-Dicer2* from BDSC. *PBac{WH}f01095* and *PBac{RB}Sin1^[e03756]^* lines used for generating a precise chromosomal deletion for the *CG5003* genomic region were obtained from the Exelixis collection at Harvard University. The deficiency line used for *nebu* testing (*Df(3L)ED4486*), the *w*; *P{His2Av-EGFP.C}2/SM6a* line (BL24163) used for live imaging crosses, and *vasa-Cas9* (BL51323) used in generating a *nebu* deletion allele were also obtained from BDSC.

### Whole ovary, mature oocyte and embryo collection

Whole ovaries were hand dissected from fattened females in Grace’s Insect Medium, unsupplemented (Life Technologies, Carlsbad, CA), and fixed in 4% formaldehyde and stained with Hoescht as in (Page and Orr-Weaver 1997). Embryos were collected for 2 hr, dechorionated in 50% bleach and washed with embryo wash buffer (0.9% NaCl, 0.03% Triton X-100). Embryos were fixed and then stained with rat anti-α tubulin YOL1/34 (AbD Serotec, Raleigh, NC) and DAPI, as described in (Shamanski and Orr-Weaver 1991). Immunofluorescence samples were scored on a Nikon ECLIPSE Ti microscope with Plan Fluor 10x or Plan Apo 20x objectives. Images were analyzed with ImageJ software.

For qRT-PCR analysis, 30 mature oocytes were collected in 15 µL medium, then frozen in liquid N_2_ in 1.5 mL tubes and stored at −80°. For glycogen and glucose measurements, 200 mature oocytes were isolated for each sample, frozen in liquid N_2_ in 1.5 mL tubes and stored at −80° until use in preparing extracts.

For live imaging, embryos were collected at 25° for 2 hr, dechorionated and mounted on a 22 × 30 mm coverslip with embryo glue and covered with a 1:1 mixture of halocarbon oil 27 and 700. Embryos were then imaged on a Nikon ECLIPSE Ti microscope with a Plan Apo 20x objective. Only embryos in which nuclei were visible were selected for imaging, with a time lapse series being acquired for subsequent divisions until cellularization. All videos were analyzed with ImageJ software.

### qRT-PCR analysis

To measure target mRNA knockdown, total RNA was isolated from whole lysates of mature oocytes by homogenizing them in TRIzol (Invitrogen) according to manufacturer’s instructions. RNA was isolated from mature oocytes dissected from females that were the progeny of the cross between males of each RNAi line and *matα4-GAL4*, *UAS-Dicer2* virgin females. The RNA was treated with a TURBO DNA-free kit (Ambion), and 500ng of the resulting RNA was used in a 20μl cDNA synthesis reaction that was performed according to manufacturer’s instructions (Promega Reverse Transcription System), using random primers. The levels of each target mRNA and an *actin5C* or *RP49* control were quantified by qPCR. Levels of mRNA were normalized to a control sample obtained from mature oocytes dissected from females that were the progeny of the cross between males of an *mCherry* RNAi line and *matα4-GAL4*, *UAS-Dicer2* virgin females.

### Precise deletion generation

To generate a precise deletion of the chromosomal region containing *CG5003* (*Df(3R)98B5-6*), we crossed a hsFLP line (BL6) to *PBac{RB}Sin1^[e03756]^*, and the resulting progeny were then crossed to *PBac{WH}f01095. hsFLP*;; *PBac{WH}f01095/ PBac{RB}Sin1^[e03756]^* female progeny were heat shocked at 37° for 1 hr, then crossed to a *w;;TM3/TM6* balancer line. Male progeny from this cross were used in single male crosses to create individual lines. Each line was then screened by PCR for the presence of the desired deletion.

### CRISPR deletion of nebu and CG7777

To generate null alleles of *nebu* and *CG7777*, we produced pU6-gRNA constructs as described in (Gratz *et al.* 2014). For deletion of *nebu*, we made two constructs expressing gRNAs targeting the following sequences (PAM is underlined): *5′-CTGTAAATTAATATCTCAAATGG-3′* and *5′-ATTGCCCCTTACTTTTCCGCGG-3′*. For deletion of *CG7777*, we made constructs expressing gRNAs targeting the following sequences (PAM is underlined): *5′-ACTGGGTGGGACCTGTGCTGGG-3′* and *5′-TTTAAGGTGCGATATTGTTTTGG-3′*. We tested all gRNAs for predicted efficiency using the Drosophila RNAi Screening Center efficiency prediction tool. To induce homology-directed repair and insert a visible selection marker, we generated a *pHD-dsRED* plasmid as described in (Gratz *et al.* 2014), with ∼1.2-1.4 kb of homology on each end starting immediately outside of the gRNA target sites. Plasmids were co-injected into embryos from *vas-Cas9* flies. All injections were performed by BestGene, Inc. services. Transformants were then screened for successful deletion of the *nebu* or *CG7777* regions by the presence of the *dsRED* marker, and the presence of the deletion was further confirmed by PCR.

### Hatching assays

For fertility testing by hatching assay, virgin females of the desired genotypes were placed in a small collection cage with wild-type males, in a 1:2 female:male ratio. Flies were kept with a molasses plate containing wet yeast, which was replaced daily. On the second, third and fourth day after collection setup, eggs were collected from the replaced plate and 100 eggs were moved to a fresh plate, and placed for 26-28 hr at 25°, after which time unhatched eggs were counted to obtain the number of eggs hatched. Each hatching assay was performed for three biological replicates (three independent crosses for the same genotypes).

### Glycogen measurements

Measurements of glycogen content in mature oocytes was performed as previously described (Sieber *et al.* 2016). Briefly, females of desired genotypes were dissected to collect 200 oocytes. The samples were then homogenized in 120 μl of 1xPBST (0.1% triton) and heat-treated for 3 min at 100°. The resulting heat-treated lysate was then centrifuged at 2500 × g for 3 min. Glycogen was then assayed using the glucose oxidase kit (Sigma, cat.# GAGO20-1kt) and amyloglucosidase (Sigma, cat.# 1602) as described in (Tennessen *et al.* 2014a). Protein levels were assayed by using the Bio-Rad protein assay reagent (cat.# 500-0006). All glycogen and triglyceride measurements were then normalized to total protein. All data presented are derived from three biological replicated samples, and each biological replicate is an average of at least 5 technical replicates.

### Data availability

Strains and plasmids generated in this study are available upon request. Note that due to difficulties in stock maintenance in recent months the *Df(3R)98B5-6* and *nebu^7^* stocks have been lost. The FRT transposon stocks used to generate this deficiency and the plasmids used for CRISPR knock out to generate the *nebu^7^* allele are available. The authors affirm that all data necessary for confirming the conclusions of the article are present within the article, figures and tables. Excel files that include the primary source data have been included in supplemental material.

In addition to the Excel source data files, the supplemental material contains three tables, four figures and legends, and a reagent table. Supplemental material available at figshare: https://doi.org/10.25387/g3.12654188.

## Results

### Targeted screen of proteins under developmental control at the oocyte-to-embryo transition

From the list of proteins whose change in levels suggest developmental regulation, we selected candidates of interest for a targeted screen using germline-specific RNAi depletion. We focused on proteins whose levels increase during oocyte maturation and then decrease upon egg activation, which could be factors involved in oocyte maturation, and on proteins whose levels remained unchanged during oocyte maturation but increased upon egg activation, which could be factors needed for the start of embryonic development. This comprised a list of 183 proteins (Kronja *et al.* 2014a). Out of this group, we limited our candidate selection to genes with unknown roles during the events oocyte-to-embryo transition. This led to a final group of 53 genes to screen (Table S1, Table S2).

We obtained all available RNAi lines targeting these genes (Table S1). We crossed each of these lines to the *matα*>GAL4 driver (Bossing *et al.* 2002), for germline-specific depletion of our candidates, also driving *UAS-Dicer2* (Dietzl *et al.* 2007) expression for knockdown enhancement. For each of our crosses, we dissected and stained ovaries from the progeny to assess morphologically the oocyte stages representing oocyte maturation, as well as collected ∼2hr embryos to examine potential defects during the early embryonic divisions. For each cross, we also evaluated the level of knockdown achieved for the target mRNA by q-RT-PCR analysis (Table S1). For candidates for which transposable element insertion alleles were available (Table S2), we examined the ovaries and embryos laid by females transheterozygous for two of these alleles for potential defects. The use of transheterozygous females avoided confusion that might result from homozygosing other secondary recessive mutations present in the chromosome. In cases in which there was only one mutant allele available, we examined the phenotype in homozygotes for the allele.

Through this approach, we identified 13 genes whose knockdown or disruption with transposable element insertion alleles resulted in an apparent morphological phenotype in either oogenesis or early embryogenesis. For nine of these genes, we could recapitulate a phenotype after two further rounds of testing. In the other four, low penetrance reduced our confidence in a defect. Ovary depletion of *mod(mdg4)* and *mei-P26* resulted in characteristic early embryonic arrest (Büchner *et al.* 2000) and developmental arrest in early oogenesis (Page *et al.* 2000; Neumüller *et al.* 2008), respectively. *mod(mdg4)* was also found through a recent genetic screen for factors involved in oogenesis, egg activation and embryogenesis (Zhang *et al.* 2018). Reduction of the activity of *carbonic anhydrase 2* (*cah2*) led to embryos exhibiting multiple arrested nuclei, though we decided to not pursue this gene because of the multiple ways that perturbing metabolism by affecting the function of this gene could indirectly impact mitosis. For the same reason, we chose not to investigate further *NaPi-III* (*CG42575)*, a gene encoding a putative sodium/phosphate symporter. We did not follow up on three genes of unknown function, *CG30377*, *CG14230* and *CG15570*, because the phenotypes indicated that earlier developmental defects indirectly affected the embryonic divisions. Embryos laid by *CG30377* mutants failed to be fertilized, whereas mutants *CG14230* and *CG15570* exhibited abnormal nurse cell numbers in some egg chambers in oogenesis. Finally, we focused our attention on the remaining two genes, *CG5003* and *CG10960*, encoding a predicted E3 ligase subunit and a putative transmembrane glucose transporter, respectively.

### CG5003 encodes an F-box protein required for early embryogenesis

*CG5003* is an uncharacterized gene encoding an F-box only protein. F-box proteins are the variable substrate adaptors for the SCF E3 ubiquitin ligase complex, mediating proteasome-dependent degradation of specific protein targets throughout the cell cycle (Petroski and Deshaies 2005; Skaar *et al.* 2013). Knockdown of *CG5003* in the germline resulted in a considerable proportion of embryos that appeared to be arrested or delayed during the first or second mitotic divisions, though no phenotype was observed during oogenesis. However, this phenotype had low penetrance and was only observed in one of four *CG5003* RNAi lines tested, although it correlated with an observed 80% *CG5003* mRNA knockdown in that line.

**Figure 1:**
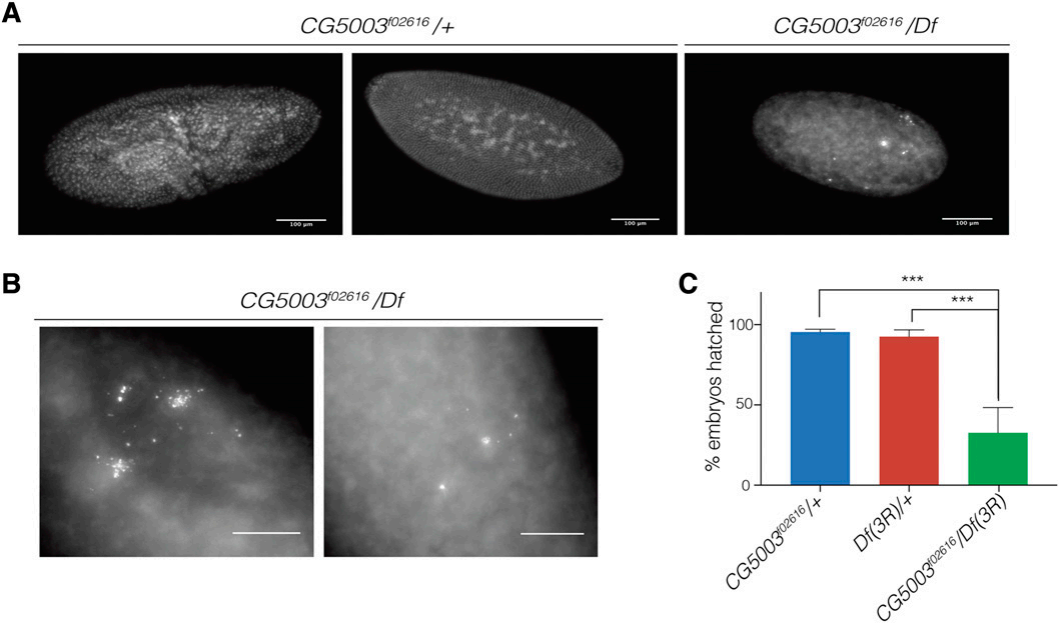
*CG5003* mutants arrest in early embryogenesis. (A) We collected 0-2 hr embryos and aged them for 2.5 additional hours, then fixed and DAPI stained them. We found that a fraction of embryos from *CG5003*^*f02616*^*/Df* mutant females were arrested prior to cellularization (25.2%, n = 131; right panel), whereas embryos from sibling control females continued development normally (97.6%, n = 147; left and middle panel). Scale bar represents 100 μm. (B) Many embryos laid by *CG5003*^*f02616*^*/Df* mutant females exhibited fragmented chromosomes. Scale bar represents 50 μm (C) The majority of embryos from mutant females for *CG5003* failed to hatch, in contrast to embryos from heterozygous females (one-way ANOVA, multiple comparisons, *** = *P* < 0.0001).

Due to the low penetrance of the embryonic-arrest phenotype observed with RNAi, we decided to examine a transposable element insertion that disrupted the *CG5003* gene. Because only one of the available insertion alleles (*CG5003*^*f02616*^) was inserted in the coding region of the gene and transposable elements inserted in the 5′ region of genes usually are hypomorphic, we used FRT-mediated recombination to generate a deficiency spanning ∼21 kilobases, completely deleting the *CG5003* coding region in addition to complete or partial deletion of eleven surrounding genes. We named this deficiency *Df(3R)98B5-6*. When crossed to *CG5003*^*f02616*^ we found that 25.2% embryos laid by *CG5003*^*f02616*^/*Df* or the *CG5003* homozygous mutant females arrested in early embryogenesis (Figure 1A), whereas 97.6% of control embryos progressed normally through development. The observed arrest in *CG5003* mutant embryos appeared to be during the first or second round of mitotic divisions, and *CG5003* mutant exhibited fragmented chromosomes (Figure 1B). Additionally, embryos laid by *CG5003* mutant females exhibited a significantly reduced hatch rate compared to *CG5003*^*f02616*^/+ and *Df/+* sibling controls (Figure 1C). The early embryonic mitotic arrest resulting from both RNAi and the transposon allele of *CG5003* over a deficiency indicates that deletion of adjacent genes is not likely responsible for the *CG5003*^*f02616*^/*Df* phenotype. Given that *CG5003* encodes a F-box protein predicted to act through SCF, this phenotype might be reflective of a role for a ubiquitin-mediated protein degradation pathway at this time.

### Mutations in nebulosa cause mitotic aberrations and a developmental delay during embryogenesis

**Figure 2:**
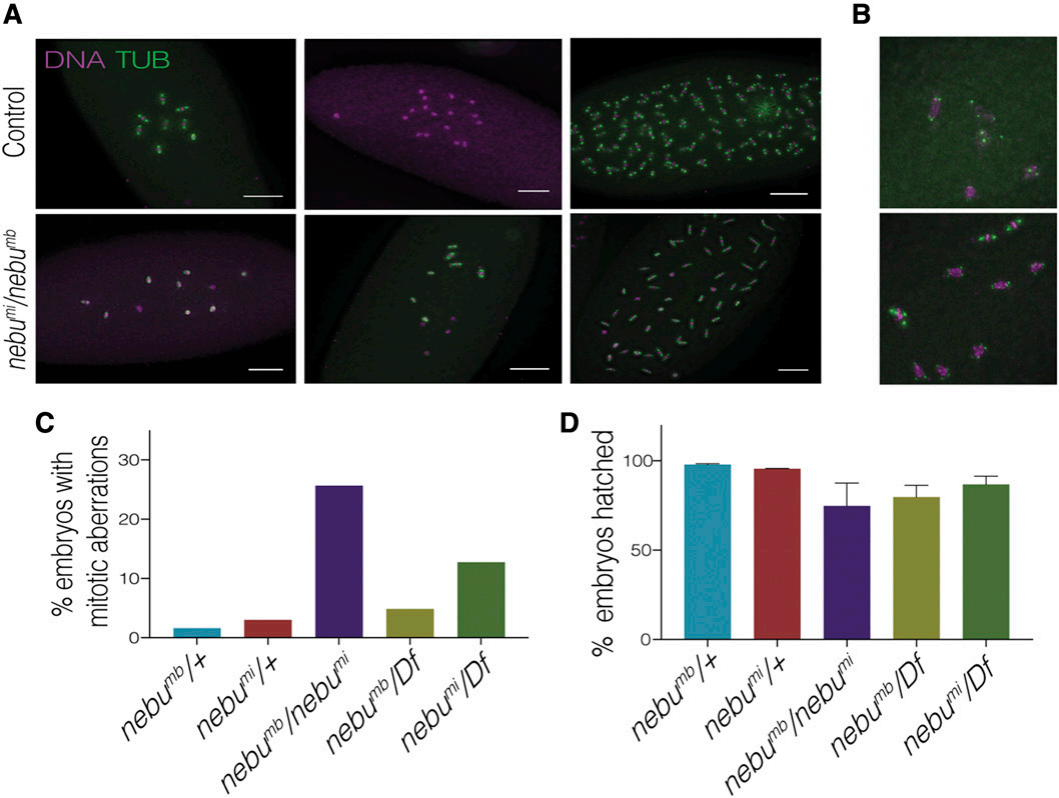
Mutations in *nebu* lead to mitotic defects in early embryogenesis. (A) Embryos from females mutant for *nebu* (*CG10960*) exhibited aberrant mitotic divisions, resulting in loss of integral nuclear doublings and abnormal mitotic spindles, such as those shown in (B). Each image represents a different embryo of the designated genotype. Scale bar represents 100 μm. DNA is shown in magenta, and tubulin in green. (C) Quantification of embryos with mitotic aberrations, like those in (A), in various *nebu* allele combinations. The difference between the observed phenotypic frequencies in *nebu*^*mi*^*/Df*, *nebu^mb^/Df* and *nebu*^*mi*^*/nebu^mb^* embryos and heterozygous control embryos was significant (Binomial test, *P* < 0.0001). *nebu*^*mi*^*/nebu^mb^* exhibited the greatest frequency of mitotic defects. Percentage values are out of total collected embryos, and a minimum of 200 embryos were collected for each genotype. *Df* refers to a chromosomal deficiency for the genomic region containing *nebu*. (D) Hatching assay of *nebu* mutants. No significant difference in fertility is observed among the different genotypes (one-way ANOVA, multiple comparisons, *P* > 0.05).

During our initial screen, we observed that disruption of the *CG10960* gene resulted in mitotic aberrations in mutant embryos. We named this gene *nebulosa* (*nebu*) due to the observed mitotic phenotype and its nebulous nature. Around 27% of embryos laid by heterozygous females for two transposable element insertion alleles *nebu^MB03129^* and *nebu^MI03549^* (hereto referred as *nebu^mb^* and *nebu*^*mi*^, respectively) exhibited defects in mitosis, compared to less than 5% observed in a sibling control (Figure 2A, C). Mitotic aberrations were observed at different mitotic division cycles in the early syncytial divisions, and included the formation of multipolar spindles, seemingly polyploid nuclei, and nuclei that appeared to be arrested rather than dividing (Figure 2B).

Interestingly, one or both of the *nebu*^*mi*^ and *nebu^mb^* alleles appear to be dominant negative alleles of *nebu*, as a higher frequency of the mitotic phenotype was observed in transheterozygotes between these two alleles than in embryos from mothers transheterozygous for either allele and a deficiency (Figure 2C). When heterozygous with the wild-type gene, the *nebu*^*mi*^ allele resulted in more mitotic defects than the *nebu^mb^* allele. Thus *nebu*^*mi*^ is likely dominant negative, a conclusion supported by its effect on glycogen levels (see below). Because transheterozygotes of *nebu*^*mi*^* and nebu^mb^* produced embryos with more mitotic defects than either over a deficiency, *nebu^mb^* may also have dominant negative effects. Although the molecular effect on gene function caused by the *nebu^mb^* and *nebu*^*mi*^ alleles remains unclear, both transposon insertions lie within the first intron of two long transcript forms and upstream of a shorter form (Thurmond *et al.* 2019). The orientation of the transposon insertions with respect to the direction of transcription of *nebu* is opposite, and this may impact how they affect gene expression. To test directly the consequence of reducing transcript levels of *nebu* we examined RNAi lines against *nebu* (Figure S1). Two lines were examined that reduced *nebu* mRNA levels to 13–20% of wild-type levels. This resulted in mitotic abnormalities in the early embryos like those observed in the embryos laid by females with the transposon insertion alleles.

Surprisingly, no significant difference in female fertility was observed in any *nebu* mutant allele combination as compared to their sibling controls (Figure 2D). The prevalence of the mitotic phenotype and absence of any effect on fertility of *nebu* mutant females suggested the possibility of some sort of developmental compensation occurring. Mechanisms for removing aberrant nuclei resulting from errors in mitotic divisions are present in the early syncytial embryo (Sullivan *et al.* 1990; Sibon *et al.* 1997, 1999; Rothwell *et al.* 1998). Nuclei that accumulate extensive DNA damage can be identified by the embryo as faulty and undergo nuclear fallout, which removes them from the blastoderm layer. These nuclei then become part of the yolk nuclei in the interior of the embryo prior to cellularization. This mechanism can result in a delay in development to allow for damaged nuclei removal or for compensatory nuclear divisions (Sibon *et al.* 1997, 1999). A developmental delay could account for the normal hatch rate in *nebu* mutants despite the dramatic mitotic phenotype.

**Figure 3:**
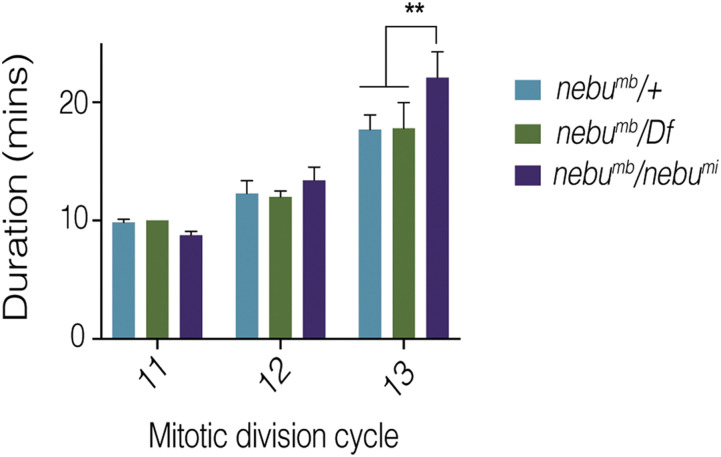
*nebu* mutants exhibit a developmental delay prior to cellularization. Live imaging quantification of embryonic division cycle times of different *nebu* allele combinations. Division cycle progression was followed by expression of an H2Av-GFP transgene marking the nuclei. Each bar represents measurements from a minimum of five embryos. A significant delay was observed during the thirteenth division cycle of *nebu*^*mi*^*/nebu^mb^* embryos (one-way ANOVA, multiple comparisons, ** = *P* < 0.001), consistent with developmental compensation of the phenotype prior to cellularization.

We evaluated the developmental timing by live imaging of *nebu* mutant embryos expressing a H2Av-GFP transgene whose expression marks nuclei throughout all stages of the division cycle. Due to the autofluorescence from the yolk that made imaging early mitotic cycles difficult, we focused on the last three cycles prior to cellularization, cycles 11, 12 and 13. We observed no significant difference in division time between embryos from *nebu^mb^/Df* *vs.* *nebu^mb^/+* mothers, but a significant delay in *nebu^mb^/nebu*^*mi*^ embryonic division time (Figure 3). This delay was observed in mitotic cycle 13, the last syncytial division prior to cellularization. These results suggest that although disruption of *nebu* results in errors in the early mitotic divisions, these can be overcome by the mechanism to delay development and allow the embryos to recover and proceed past the cellular blastoderm stage.

### Deletion of nebulosa results in female infertility and an increase in yolk nuclei number

Although we observed embryonic nuclear division defects in the transposable element insertion mutants for *nebu* and when mRNA levels were reduced by RNAi, neither the transposon alleles or the levels of transcript depletion were expected to result in complete loss of *nebu* gene function. Therefore, we generated a complete *nebu* loss-of-function by deleting the *nebu* gene using CRISPR. We designed gRNAs targeting the ends of the *nebu* genomic locus to remove this region completely without affecting any of the nearby genes (Figure S2A). Our strategy resulted in a successful precise *nebu* deletion allele, *nebu^7^*, which we could then use in combination with a chromosomal deficiency to obtain adult flies in which no *nebu* function was present (Figure S2B).

**Figure 4:**
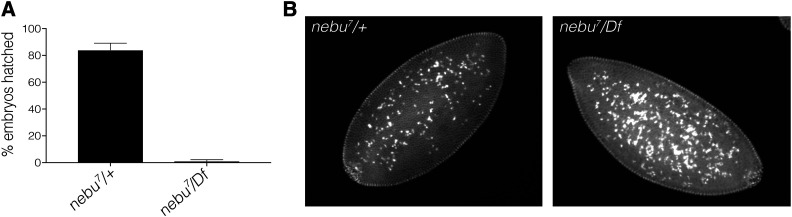
Complete loss of *nebu* function leads female infertility and increased yolk nuclei in embryos. (A) Hatching assay analysis of eggs laid by *nebu* null females. Eggs laid by *nebu^7^/Df* females showed a significant decrease in hatching (two-tailed *t*-test, *P* < 0.0001) as compared to a sibling control. *nebu^7^* is a CRISPR null allele of *nebu*. (B) An increase in yolk nuclei was observed in blastoderm *nebu^7^/Df* embryos (57%) as compared to a stage-matched sibling control embryos (7%). Embryos were collected for 2 hr and aged for 1 hr, then fixed and stained with DAPI.

In contrast to the *nebu* transposable element insertion mutants, we found that deletion of *nebu* led to female infertility and a recessive maternal-effect embryonic phenotype. Embryos laid by *nebu^7^/Df* females failed to hatch, whereas the majority of *nebu^7^/+* embryos did (Figure 4A). To our surprise, embryos from *nebu^7^/Df* mothers did not exhibit the mitotic phenotype observed in the transposable element insertion mutants or in the RNAi lines. These embryos appeared to progress normally through the early embryonic mitotic cycles. However, we observed a higher frequency of embryos with increased yolk nuclei number in embryos from *nebu^7^/Df* females (53%) than sibling controls (7%) (Figure 4B), which suggests the presence of increased mitotic errors in these mutants. Given *nebu* null mutants failed to hatch, this phenotype could be indicative of underlying errors that lead to lethality in later embryonic stages.

### nebulosa encodes a putative sugar transporter with two different protein isoforms

**Figure 5:**
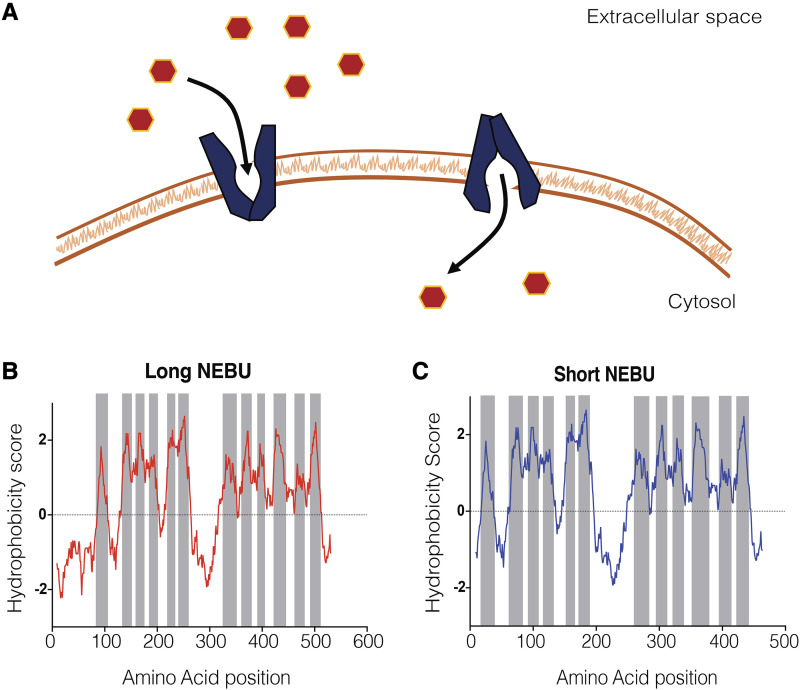
*nebu* encodes a putative transmembrane sugar transporter. (A) Schematic of a transmembrane sugar transporter, of the SLC2 superfamily, mediating transport across the plasma membrane. SLC2 family transporters are facilitative transporters and can transport a variety of different sugar substrates. These types of transporters can also mediate transport bidirectionally and between intracellular compartments and the cytoplasm. (B and C) Hydrophobicity analysis of NEBU protein sequence using the Kyte and Doolitle algorithm (Kyte and Doolittle 1982), reveals twelve predicted transmembrane domains. (B) Hydrophobicity plot of long NEBU, which has an extended N-terminal region containing a dileucine motif, an intracellular retention signal. (C) Hydrophobicity plot of short NEBU, which lacks the extended N-terminal region and is predicted to be localized at the plasma membrane.

*nebu* is predicted to encode a sugar transporter from the SLC2 superfamily of transmembrane transporters (Figure 5A) (Thurmond *et al.* 2019). *In silico* analysis of NEBU reveals the predicted presence of the characteristic major facilitator superfamily domain, containing twelve transmembrane regions characteristic of this family of transporters (Uldry and Thorens 2004; Mueckler and Thorens 2013). *nebu* has three different mRNA forms that are predicted to be translated into two distinct protein isoforms, short-NEBU and long-NEBU, differing only in an N-terminal cytoplasmic domain (Figure 5B, C) (Thurmond *et al.* 2019). One of these isoforms, long-NEBU, contains a di-leucine motif ([DE]XXXL[LI]) that is known to act as an internalization signal (Aerni-Flessner *et al.* 2011; Mueckler and Thorens 2013). This suggests that whereas short-NEBU could be localized to the plasma membrane, the long-NEBU isoform could act in transport between intracellular compartments. No prediction can be made about the substrate specificity of either isoform.

### Glycogen levels are affected in nebu mutants

**Figure 6:**
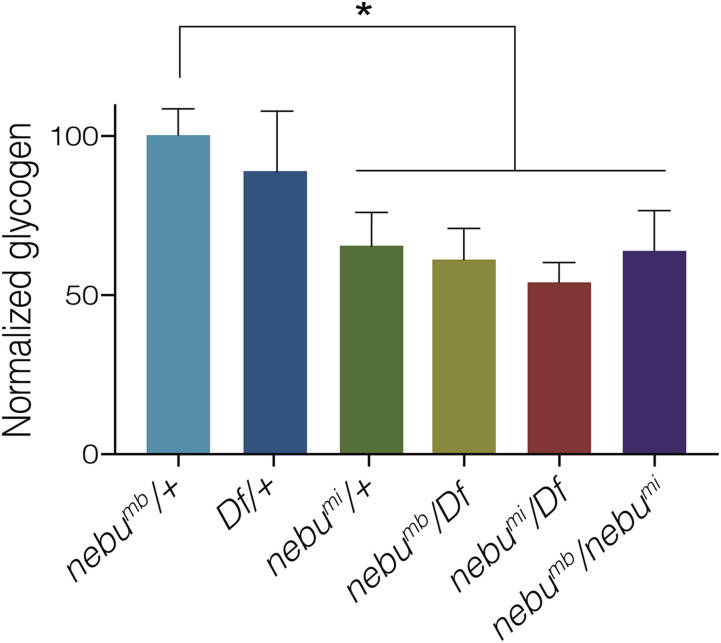
Glycogen levels are reduced in *nebu* mutant mature oocytes. Colorimetric glycogen levels were measured from isolated mature oocytes of the designated genotype. Five technical replicates were performed for each sample. Each bar represents average measurements for five biological replicates, and error bars correspond to standard deviation. A significant decrease in glycogen was observed between sibling controls, *nebu^mb^/+* and *Df/+*, and *nebu* mutants of various allele combinations (one-way ANOVA, multiple comparisons, * = *P* < 0.05). However, no significant difference was observed between *nebu* mutants and the *nebu*^*mi*^*/+* control, consistent with this allele having dominant negative properties.

During oocyte maturation, carbohydrates are accumulated in the form of glycogen (Sieber *et al.* 2016). These stores of glycogen are used as an energy source later in embryogenesis. Because sugar transport across membranes can provide substrates for sugar metabolic processes in the cell, including glycogen metabolism, it is possible that NEBU could be part of the glycogen accumulation pathway during late oogenesis. If this were the case, we would expect altered levels of glycogen in *nebu* mutant mature oocytes. Using a colorimetric assay, we measured glycogen levels in mature oocytes from females with different *nebu* allele combinations. We observed that relative to *nebu^mb^/+* controls, glycogen levels were reduced in all *nebu* mutant oocytes but not in *Df/+* oocytes (Figure 6). This is consistent with NEBU function being needed for the glycogen accumulation that occurs during late oocyte maturation, perhaps by transport of substrates from outside the cell or between cellular compartments. One of the controls, *nebu*^*mi*^*/+*, showed no significant difference in glycogen levels compared to *nebu* mutants but significantly lower levels when compared to the other controls (Figure 6). This is consistent with the *nebu*^*mi*^ allele being a dominant negative mutant of *nebu*, as indicated by the embryonic phenotype.

As mutations in *nebu* lead to defects in mitosis and arrest in embryogenesis, and *nebu* mutant oocytes showed defects in glycogen accumulation, we wondered whether disrupting enzymes involved in glycogen metabolism could lead to a similar embryonic phenotype (Table S3). Glycogen accumulation in cells involves a series of enzymatic reactions catalyzed by a defined group of enzymes (Figure S3A). We depleted enzymes from the glycogen pathway by germline-specific RNAi and evaluated the resulting embryonic phenotype. We observed that RNAi against most targeted enzymes resulted in no significant difference in embryonic phenotype as compared to an *mCherry* RNAi control (Figure S3B). Knockdown of *GlyP*, however, resulted in an increase in embryos with mitotic defects, which approximated that observed in *nebu* RNAi (Figure S3B). *GlyP* encodes for glycogen phosphatase, the enzyme that catalyzes the first step in glycogen breakdown.

### CG7777, a putative aquaporin, is dispensable for female fertility

In addition to selecting genes without regards to function for our screen, we also selected candidates whose function indicated they should be critical for the oocyte-to-embryo transition. Hydration and swelling of the oocyte occur during egg activation in Drosophila (Horner and Wolfner 2008b; Avilés-Pagán and Orr-Weaver 2018). This hydration precedes Ca^2+^ influx into the oocyte, and the accompanying swelling might represent a mechanism to create osmotic pressure that serves as a form of mechanical stimulation of the oocyte (Horner and Wolfner 2008a). The *CG7777* gene encodes a putative aquaporin, a member of a family of proteins that facilitate passive transport of water molecules across the plasma membrane. CG7777 protein levels are increased during oocyte maturation and are decreased after egg activation (Kronja *et al.* 2014a). Given the importance of hydration during egg activation, this predicted that *CG7777* function could be critical at this time. We thus investigated a potential role for *CG7777* during egg activation.

As we found no effect from germline-specific depletion of *CG7777* mRNA by RNAi (Table S1), we generated a knockout allele of the *CG7777* gene using CRISPR. Two guide RNAs were designed to target the coding region of *CG7777* and generate a complete deletion by homology-directed repair with a donor plasmid carrying a *dsRED* cassette flanked by sequences with homology to the *CG7777* genomic flanking sequences (Figure S4A). We visually screened for the presence of the *dsRED* marker, and confirmed the presence of the desired deletion by PCR (Figure S4B).

We found no significant differences between oocyte morphology and progression through meiosis in *CG7777*^*del*^ homozygotes or *CG7777*^*del*^/*Df* as compared to heterozygous control. Despite the lack of a morphological phenotype, we observed a small but significant decrease in the hatch rate of eggs laid by *CG7777*^*del*^ homozygous females when compared to *CG7777*^*del*^ heterozygous females (Figure S4C). However, we detected no significant difference between *CG7777*^*del*^ homozygotes and *CG7777*^*del*^*/Df* or *Df/+*, suggesting that the effect on fertility of *CG7777*^*del*^ homozygous females may be due to background mutations on the chromosome. Thus we conclude that CG7777 is not required for egg activation and female fertility. There may be redundancy in function between CG7777 and other members of the aquaporin family of proteins present in the oocyte, which could account for the lack of an observed phenotype. A more extensive analysis of aquaporin gene mutants will be needed to better understand the role of this protein family, and hydration, during egg activation.

## Discussion

The lack of transcriptional activity in the oocyte during late oogenesis means that remodeling of the proteome is crucial for the proper transition from oocyte to embryo. Thus, key regulators must be controlled by posttranslational modifications or control of protein levels. Although recent studies have addressed posttranslational modifications to understand the regulation of known players and to screen for potential new regulators of the oocyte-to-embryo transition (Krauchunas *et al.* 2013; Guo *et al.* 2015; Zhang *et al.* 2018), we instead focused on changes in protein levels across the transition. We reasoned that changes in protein levels could be diagnostic of proteins with pivotal roles during the events of oocyte maturation and egg activation in Drosophila. Indeed, many known key regulators, such as GNU, LID and DHD, exhibit protein level patterns that are consistent with their functions during the transition (Torres-Campana *et al.* 2020; Kronja *et al.* 2014a; Petrova *et al.* 2018). Similar to these proteins, many other proteins show protein level patterns suggestive of developmental control (Kronja *et al.* 2014a).

Out of 53 genes that we screened based on protein level patterns, we identified several that have potential roles during the events of the oocyte-to-embryo transition. For many of these genes we observed no phenotype upon mRNA depletion. Although we cannot exclude the possibility that these genes are not essential for the transition, an alternative explanation is that the achieved mRNA depletion was not enough to reduce levels below the threshold required for gene function. For many of the genes in our screen, limited resources for their disruption were available, and these could have given an insufficient reduction in function to observe a phenotype. For example, driving RNAi of *CG17018* with matα>GAL4 did not give a phenotype, but it recently has been shown to be required for the meiosis I to II transition and named *meiosis arrest female 1* (*MARF1*) (Kawaguchi, Ueki, and Kai 2020). It is also possible there are redundant functions between these genes and others that prevented us from observing a phenotype. Moreover, because our screening of phenotypes was based primarily on morphological analysis, phenotypes that resulted in molecular changes without a visible phenotype would have been missed under our approach. Despite these caveats, this screen led to the to the identification of several genes with potential roles during the oocyte-to-embryo transition in Drosophila, including two previously uncharacterized genes, *CG5003* and *nebu*.

The demonstration of an embryonic requirement for a component of an E3 ubiquitin ligase complex is consistent with the crucial changes to the proteome after egg activation (Kronja *et al.* 2014a). *CG5003* is predicted to encode an F-Box protein, a class of proteins that are the substrate specificity subunits of SCF complexes (Skaar *et al.* 2013). The embryonic phenotype resulting from the lack of *CG5003* function suggests the presence of an SCF^5003^ complex with specific targets to allow progression past the first few rounds of embryonic divisions. Given that not all *CG5003* embryos arrest, it is likely that other SCF complexes or different E3 ubiquitin ligases are acting at this time. Each complex could have a preference but not absolute specificity for controlling the degradation of a specific set of substrates during early embryogenesis. They also could act in concert with the protein degradation and cell cycle control mediated by APC/C complexes. It will be interesting to learn how many of the proteins that decrease in levels upon egg activation (Kronja *et al.* 2014a) are degradation targets of SCF^CG5003^ complexes. A quantitative proteomic analysis of *CG5003* mutant embryos can serve to identify which proteins are not properly degraded in these mutants compared to wild type and are thus potential targets of degradation via CG5003. These targets could then be confirmed by further analysis, such as with *in vitro* ubiquitination assays. It also remains to be tested whether *CG5003* has additional roles later than the observed arrest in the early embryonic divisions, perhaps in maternal proteome clearance after zygotic genome activation. A precedent for this derives from a recent study that identified an E2 and an E3 ligase responsible for the degradation of maternal RNA binding proteins at the maternal-to-zygotic transition (Zavortink *et al.* 2020). Experiments addressing the diversity of E3 ubiquitin complexes and the subsets of targets under their regulation in early embryos should yield new insights into proteome remodeling during embryogenesis.

Many transmembrane proteins exhibit dynamic changes in protein levels during the oocyte-to-embryo transition in Drosophila (Kronja *et al.* 2014a). However, a functional characterization of a potential role for these transmembrane transporters was lacking prior to this work. Our screen led to the identification of a role for a transmembrane sugar transporter, NEBU, during the oocyte-to-embryo transition. Deletion of the *nebu* gene does not cause lethality, and thus it appears to be essential only at the onset of development. The transporter encoded by *nebu* has homology to transporters of the SCL2 superfamily, which includes highly conserved facilitative glucose transporters with important roles in sugar metabolism across tissues (Mueckler and Thorens 2013). Although NEBU has the highest sequence similarity to GLUT6 and GLUT8, the substrate specificity for NEBU cannot be ascertained from protein sequence alone. Apart from transporting glucose, the SLC2 family of transporters can also mediate transport of other small sugars (Mueckler and Thorens 2013), so the substrate specificity of NEBU will have to be determined experimentally in the future. The observation that *nebu* mutant mature oocytes have altered levels of glycogen accumulation suggests a function for *nebu* in the glycogen accumulation pathway active during late oogenesis (Sieber *et al.* 2016). Moreover, because there are two NEBU isoforms, it is possible that NEBU has two distinct transport activities, for example, one across the plasma membrane and one between intracellular compartments and the cytoplasm.

Identification of the substrates and intracellular distribution of NEBU will help to provide a mechanistic understanding of the complexity of the *nebu* allele phenotypes and the relationship to glycogen synthesis and degradation. It remains to be clarified why the apparent partial loss-of-function transposon insertion alleles and RNAi cause an earlier mitotic arrest in the embryo than the CRISPR knock-out allele. Perhaps there is dominant suppression by hemizygosity for one of the genes within the deletion used to analyze the phenotype of the *nebu^7^* allele. Molecular analysis of the transposon insertion alleles may shed light on the basis for their dominant negative properties. In addition to these genetic puzzles, the relationship between the biochemical activity of NEBU and glycogen levels requires elucidation, given that reduction of glycogen phosphatase activity, which is predicted to increase levels of glycogen, results in the same embryonic mitotic phenotypes as *nebu*. Glycogen levels may need to be within a critical window for accurate embryogenesis. RNAi against glycogen synthetase and other genes in the glycogen pathway did not result in a phenotype, but this may be due to insufficient depletion of the mRNA.

Although it is known that glycogen is used during embryogenesis for energy (Tennessen *et al.* 2014b; Sieber *et al.* 2016; Yamada *et al.* 2019), it is unclear at what point the embryo starts relying on the stored glycogen. Our results lead to the idea that these glycogen stores could start being utilized very early in the embryo to fuel progression through early embryogenesis. Given the energy requirements of mitotic divisions (Salazar-Roa and Malumbres 2017), lowering these stores by disrupting glycogen accumulation or breakdown could lead to the embryonic mitotic defects observed in *nebu* embryos. It still remains unclear exactly how the mitotic defects relate to glycogen metabolism, and the role of sugar transport in this process. However, because glucose transport has been reported in mammalian oocytes (Wang *et al.* 2012) and loss of *nebu* results in infertility, a more detailed characterization of *nebu* is likely to yield new insights into conserved mechanism of metabolic remodeling in oocytes across species.

Investigation of proteins based solely on their changes in levels during oocyte maturation and egg activation, without regard to predicted function, uncovered the essential roles of an F box protein and sugar transporter in the oocyte-to-embryo transition. In contrast, analysis of a protein whose predicted function and expression pattern strongly implicated it in being essential for egg activation, the aquaporin GC7777, in fact did not find it to be required. Aquaporin function could be essential for the oocyte-to-embryo transition, with other family members playing a redundant role to CG7777.

Overall, our study complements recent studies aimed at identifying regulators of the oocyte-to-embryo transition in Drosophila and other organisms (Guo *et al.*, 2015; Zhang *et al.*, 2018). All of these studies have not only led to the discovery of new regulators, but have served to highlight important cellular and molecular processes during this transition. However, even with the new information obtained by these studies, a comprehensive list of regulators and the relationships between the different pathways acting at this time remains elusive. Future work aimed at uncovering additional regulators and further characterizing previously known regulators will be crucial in understanding the requirements for a proper transition from oocyte to embryo.
